# Acupuncture for Anxiety, Depression, and Sleep in Veterans with Combat-Related Posttraumatic Stress Disorder: A Randomized Controlled Trial

**DOI:** 10.3390/jcm14103443

**Published:** 2025-05-15

**Authors:** An-Fu Hsiao, Jennifer Lai-Trzebiatowski, Tyler Smith, Teresa Calloway, Chelsea Aden, Tanja Jovanovic, Besa Smith, Kala Carrick, Andrea Munoz, Megan Jung, Michael Hollifield

**Affiliations:** 1 Tibor Rubin VA Medical Center, 5901 E. 7th St, Long Beach, CA 90822, USA; jennifer.lai@va.gov (J.L.-T.); teresa.calloway2@va.gov (T.C.); chelsea.aden@va.gov (C.A.); krjcarrick@gmail.com (K.C.); andrea.munoz@va.gov (A.M.); megan.jung@va.gov (M.J.); michael.hollifield@va.gov (M.H.); 2 Department of Medicine, Health Policy Research Institute and General Internal Medicine, University of California Irvine, 100 Theory, Suite 110, Irvine, CA 92697, USA; 3 Analydata, 3835 Centraloma Drive, San Diego, CA 92107, USA; tylersmith@analydata.com (T.S.); besasmith@analydata.com (B.S.); 4 National University, 9388 Lightwave Ave, San Diego, CA 92237, USA; 5 Department of Psychiatry and Behavioral Neurosciences, School of Medicine, Wayne State University 3901 Chrysler Service Drive, Detroit, MI 48201, USA; tjovanovic@med.wayne.edu; 6 Department of Psychiatry and Behavioral Sciences, George Washington School of Medicine & Health Sciences, Washington, DC 20037, USA

**Keywords:** posttraumatic stress disorder, anxiety, depression, insomnia, acupuncture, clinical trial

## Abstract

**Objective**: Current interventions for anxiety, depression, and insomnia are efficacious, yet effectiveness may be limited by side effects and/or high withdrawal rates. Other desirable treatment options are needed. Many veterans and civilians are turning to acupuncture as an emerging therapy. Our objective was to conduct a more definitive study comparing verum with sham acupuncture (minimal needling). **Methods**: A two-arm, single-blinded randomized controlled trial (RCT) hypothesizing that both verum and sham acupuncture are effective and the effects of verum are superior to those of sham acupuncture. We recruited subjects from a single outpatient-based site, the Tibor Rubin VA Medical Center, Long Beach, CA, USA. A total of 93 treatment-seeking combat Veterans with posttraumatic stress disorder (PTSD), aged 18–55, were allocated to groups by adaptive randomization, and 71 participants completed the intervention protocols. Verum and sham were both offered as 1 h sessions, twice a week, and participants were allowed 15-weeks to complete up to 24 sessions. This was a secondary analysis from a larger study about the efficacy of acupuncture for PTSD. Outcomes for the current study were pre- to post-intervention change in the Hamilton Anxiety Rating Scale, Beck Depression Inventory, and Pittsburgh Sleep Quality Index. Outcomes were assessed pre-, mid-, and post-treatment. General Linear Models comparing within- and between-group results were analyzed in both intention-to-treat (ITT) and treatment completer models. **Results**: In total, 85 males and 8 females, with a mean age of 39.2 (median = 37.0), were randomized. For anxiety, the verum acupuncture showed a large treatment effect (*d* = 1.3), whereas sham acupuncture showed a moderate effect (*d* = 0.9). There was no statistical difference between the verum and sham acupuncture groups. Similar effects were found for depression and insomnia symptoms. Withdrawal rates were low. **Conclusions**: Both verum and sham acupuncture were efficacious in the treatment of anxiety, depression, and insomnia in a population of veterans with PTSD. However, there was no clinical difference between the verum and sham acupuncture groups. These data build on extant literature and suggest that further research on the clinical implementation and durability of acupuncture for anxiety, depression, and insomnia is warranted.

## 1. Introduction

Complementary and integrative health (CIH) therapies, including acupuncture, massage therapy, yoga, herbs and supplements, and other modalities, are highly sought out by both veterans and civilians because of their holistic healing and perceived safety [1,2]. Therefore, many veterans with psychiatric disorders, including anxiety, depression, insomnia, and posttraumatic stress disorder (PTSD), have sought acupuncture treatment as an adjunctive or alternative to pharmacotherapy and psychotherapy [3,4]. In 2011, The VHA (Veteran Health Administration) embraced the “Whole Heath” health paradigm and added eight CIH therapies, including acupuncture, to its list of medical treatments for veterans who are interested in using CIH therapies in addition to conventional medicine to treat their physical and mental conditions [5].

Evidence for acupuncture’s effectiveness for anxiety, depression, and insomnia has been mixed and weakened by a lack of design rigor in clinical trials, the heterogeneity of controls in research from inert placebos to active and wait list controls, and vague reporting or rationale for treatment [6,7]. However, recent studies have been more promising, especially when specific clinical symptoms were targeted. Within the anxiety literature, Amorim and colleagues [8] investigated the efficacy of electroacupuncture (EA) and manual acupuncture in participants diagnosed with anxiety and found that both groups exhibited improvement in symptoms both mid-treatment and post-treatment. One literature review of acupuncture for preoperative anxiety yielded significant reductions in state and trait anxiety [9], while significant differences in the reduction of anxiety symptoms were reported between sham and acupuncture groups at the end of follow-up among participants with Parkinson’s disease [10]. Yin and colleagues [11] compared the effects of EA against sham acupuncture and standard of care for depression and reported a greater reduction in depression, sleep, and anxiety symptoms at the end of treatment compared to both sham acupuncture and a waitlist control. Liu et al. [12] yielded similar findings in chronic insomnia patients, with significantly lower sleep, anxiety, and depression symptomatology in an acupuncture group compared to a sham group at follow-up.

The first published RCT of acupuncture for PTSD showed efficacy equivalence between acupuncture and group CBT and superiority to a waitlist control on self-reported symptom reduction [13], with effect retention for 3 months [14]. In this same study, which was specifically designed as a PTSD clinical trial, acupuncture also showed significant effects on depression, anxiety, and sleep symptoms compared to the waitlist control group, although the treatment effect size was less than for PTSD. Engel [15] also found significantly greater reductions in depression symptoms for PTSD patients in an acupuncture treatment group compared to usual PTSD care. We recently completed and published a study that showed verum acupuncture was more effective than sham acupuncture in reducing the psychobiology of PTSD in combat veterans [16].

The current study was aimed at replicating findings from Hollifield and Engel’s studies, indicating that in a study designed as an intervention for PTSD, acupuncture would also have therapeutic effects on anxiety, depression, and insomnia. Post hoc analyses were conducted on data from this current randomized clinical trial, which was designed to address methodological limitations by comparing verum (active) vs. sham (placebo) acupuncture in an adequately powered sample (https://clinicaltrials.gov/study/NCT02869646?term=NCT02869646&rank=1, accessed on 30 October 2024). The hypothesis was that both verum and sham acupuncture will be effective in reducing these symptoms in veterans with PTSD, with verum acupuncture being more effective than sham acupuncture in symptom reduction.

## 2. Methods

### 2.1. Trial Design, Participants, and Setting

This was a two-arm, parallel-group, single-blind, superiority hypothesized prospective RCT in treatment-seeking combat Veterans with chronic PTSD at the Tibor Rubin VA Medical Center in Long Beach, CA. Ninety-three were randomized to adequately power the study on the primary hypothesis that acupuncture is efficacious for PTSD. No extant data were available for a priori sample size/power estimates on the secondary hypotheses about anxiety, depression, and insomnia. A description of study methods has been published [17].

### 2.2. Recruitment, Inclusion/Exclusion Criteria, and Withdrawal Rules

Recruitment was from April 2018 to May 2022 using flyers and email. The inclusion criteria were veterans between the ages of 18 to 55 meeting diagnostic criteria per the Diagnostic and Statistical Manual (DSM-5) for PTSD, with a severity score of >26 on the Clinician Administered PTSD Scale-5 (CAPS-5). Common comorbidities (e.g., anxiety and depression) were allowed. Participants had 15 weeks to complete 24 sessions, could be receiving supportive care that is not evidence-based treatment for PTSD, and were not discontinued for proceeding at a slow pace. A serious adverse event required withdrawal. Those withdrawn were not replaced.

The exclusion criteria included characteristics that are known PTSD treatment confounds, might affect biological assessment, indicate past non-adherence or treatment resistance, or indicate a risk of harm [17]. The study was approved by the local VA IRB and written informed consent was obtained.

### 2.3. Randomization and Blinding

Adaptive randomization was used to ensure balance between groups on known prognostic factors (i.e., combat exposure, pre-deployment preparedness, and sex) [18,19]. One research coordinator (RC) assigned group allocation by consecutive study ID numbers. Other study personnel were blinded to ID by group. These kept assessors blinded to allocation, and investigators and clinicians blinded to assessment data. The participants were blinded to allocation. However, the acupuncturist could not be blinded in this single-blind trial design.

### 2.4. Outcomes and Measures

#### 2.4.1. General Approach

The primary hypothesis for the parent study was that the effect of verum acupuncture (ACU) on PTSD symptom severity reduction would be large and superior to that of sham needling (SHAM), as assessed by CAPS-5 [20], the gold standard assessment for PTSD. Each of the 20 items are rated from 0 (absent) to 4 (severe), summed into a single severity score. The more complete CAPS-5 scoring rules applied in this study have been published [17]. Twenty assessments were co-rated from audio-tape recordings, with an inter-rater reliability (Pearson’s r) on symptom severity of 0.91, *p* < 0.001.

#### 2.4.2. The Hamilton Anxiety Rating Scale (HAM-A)

The HAM-A was used to measure the severity of anxiety symptoms. The participants were asked to rate 14 items on a Likert scale (0–4), where <17 indicates mild anxiety, 18–24 indicates mild to moderate anxiety, 25–30 indicates moderate to severe anxiety, and >30 indicates severe anxiety [21].

#### 2.4.3. Beck Depression Inventory—II (BDI-II)

BDI-II is a widely used 21-item self-report questionnaire assessing the severity of depressive symptoms with high internal consistency [22].

#### 2.4.4. Pittsburgh Sleep Quality Index (PSQI)

PSQI is a self-rated questionnaire assessing sleep quality and disturbances over a 1-month time interval. The PSQI consists of 19 individual items that generate 7 component scores, whose sum yields a global score with a possible range of 0–21 [23].

#### 2.4.5. Safety Assessment

The safety assessment consisted of screening for suicidal and homicidal ideation and anger/irritability at regular timepoints using items from the Aggression Questionnaire and the BDI-II [24,25]. A qualified clinician provided additional screening and evaluation, as needed. Adverse (AE) and serious adverse (SAE) events were assessed at each study visit.

### 2.5. Interventions

Needles of identical diameters were used for ACU and SHAM. Differences by group, discussed below, are previously published [17].

Experimental Group: Verum Acupuncture (ACU):

Individual sessions were offered twice weekly, aiming for 24 sessions in a 15-week period. Sessions reflected clinical practice with an interview (10 min), pulse/tongue observation (5 min), standard needling to elicit DeQi (fullness, heaviness, aching, tingling but not sharp pain) (10 min), needle retention (30 min), and removal and conclusion (5 min). The participants received a standard point prescription designed in previous work translating PTSD symptoms into Traditional Chinese Medicine (TCM) diagnostic patterns [14,25]. The sessions alternated between supine (11 points) and prone (14 points) positions to avoid point fatigue (tolerance from frequent use). In addition to standard points, up to 3 discretionary points from a list of 15 were chosen at each session, addressing transient presentation or augmentation to primary patterns. Electric stimulation was delivered during needle retention across two-point pairs during both supine and prone positions at a mixed frequency of 2/100 Hz.

Placebo Control Group: Minimal Needling (SHAM):

The individual sessions were the same in terms of time, frequency, and duration as ACU. Three elements defined SHAM in this study: (1) location 2 cm lateral or medial to reference points, which were not expected to affect PTSD symptoms, (2) superficial insertion (<0.25 inch), and (3) the relative absence of stimulation due to shallow insertion and the use of a sham electro-stimulator (blinking light without current). The actual distance between the acupuncture reference point and sham point was approximate, with consideration given to (1) nearby acupuncture meridians, (2) superficial or deep anatomical features (e.g., visible vessels), and (3) individual body proportions (#2 and #3 are also relevant for ACU). The acupuncturist did not manipulate or obtain DeQi, and needles were only adjusted more superficially to minimize reported sensations (e.g., stinging/irritation). The protocol also used alternating 11 front and 14 back points and provided sham-electrical stimulation across two-point pairs.

To assess protocol adherence and the validity of blinding, all sessions were videorecorded. One reviewer viewed/analyzed 10% of the sessions (about 320 h) and remained blinded to grouping. Both ACU and SHAM sessions had high adherence rates (98%) to protocol points. No participant was heard commenting about the intervention they thought they were getting.

### 2.6. Data Management and Analyses

Descriptive and univariate analyses of the participants’ characteristics by group status were completed using Pearson chi-square tests of association. Matched paired *t*-tests, Cohen’s d, and repeated measures MANOVA for within and between participants’ differences over time were calculated for both intention-to-treat (ITT) and participants who completed (TC). A Generalized Linear Model leveraging Generalized Estimating Equations for correlated outcome data was developed to test differences in diagnosis over time. Statistical analyses were conducted with SAS. 9.4 [26].

### 2.7. Ethics and Safety

This study was approved by the Institutional Review Board, VA Long Beach, and monitored by the VA Data Monitoring Committee.

## 3. Results

### 3.1. Demographics, Clinical Characteristics, and Study Flow

**Figure 1:**
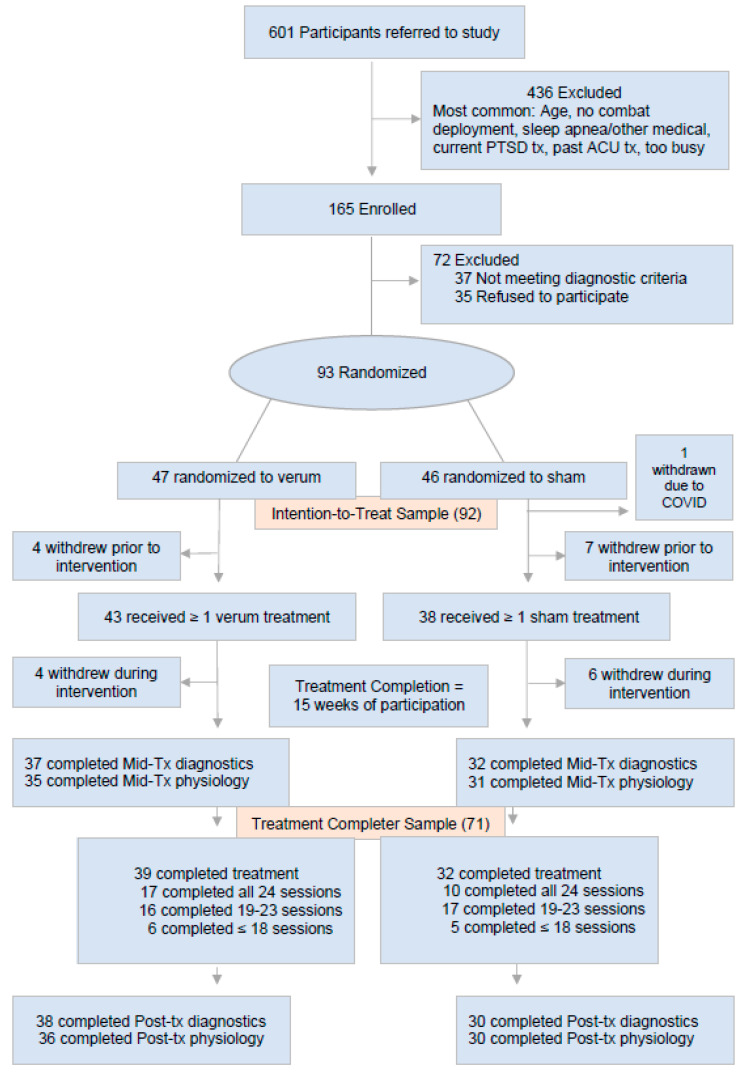
Participant Recruitment Flow Diagram.

Allocation provided group balance on demographics and clinical characteristics, particularly on controlled variables (Table 1). Figure 1 shows study flow: 601 were referred, 165 enrolled, 93 were randomized, and 71 completed interventions. One participant was withdrawn due to COVID; their data were excluded from the analyses.

The baseline CAPS-5 for verum acupuncture was 37.1, and that for sham acupuncture was 36.6. The baseline HAM-A for verum acupuncture was 33.6 and 32 for sham acupuncture. The BDI for verum acupuncture was 32 for verum and 30.7 for sham. Similarly, the PSQI values for verum and sham acupuncture were both 14.2.

Criterion A events were direct combat for 72 (77.4%), another violence or loss event for 10 (10.8%), sexual assault for 7 (7.5%), and accident for 4 (4.3%). Study retention was similar between groups: after randomization, it was 77.2% for all (83.0% for ACU, 71.1% for SHAM), and after >1 treatment session, it was 87.7 for all (90.7% for ACU, 84.2% for SHAM).

### 3.2. Primary Endpoints

Hamilton Anxiety Rating Scale (HAMA) Severity (Table 2).

- Intragroup Analyses

The intent to treat (ITT) analysis (N = 93) showed a large effect size of verum pre- to post- (Δ = −14.1; *t* = 8.3; *p* =< 0.0001; *d* = 1.3). The sham pre- to post- analysis had a smaller effect size (Δ = −8.9.1; *t* = 5.3; *p* =< 0.0001; *d* = 0.9). Similarly, the TC analysis (N = 71) also showed a larger effect size of verum pre- to post- (Δ = −14.6; *t* = 8.9; *p* =< 0.0001; *d* = 1.4) compared to sham (Δ = −9.9.1; *t* = 6.3; *p* =< 0.0001; *d* = 1.15).

- Intergroup Analyses

In contrast to intragroup analyses, the ITT (N = 93) intergroup analyses comparing sham vs. verum showed a small effect size and was not statistically significant at mid-treatment (Δ = −0.36; *t* = 0.14; *p* = 0.89; *d* = 0.03). The intergroup analyses comparing sham vs. verum also showed a small effect size and was not statistically significant at the end of treatment (Δ = −3.8; *t* = 1.43; *p* = 0.16; *d* = 0.34). The intergroup, TC analyses (N = 71) also showed a small effect size and was not statistically significant at both mid-treatment (Δ = −0.21; *t* = 0.08; *p* = 0.94; *d* = 0.02) and the end of treatment (Δ = −4.0; *t* = 1.47; *p* = 0.15; *d* = 0.36)

Beck’s Depression Inventory Scores (BDI) (Table 3).

- Intragroup Analyses

The ITT analyses (N = 93) showed a large effect size for verum pre- to post- (Δ = −13.2; *t* = 7.8; *p* =< 0.0001; *d* = 1.26), while sham had a smaller effect size (Δ = −11.5.1; *t* = 6.01; *p* =< 0.0001; *d* = 0.93). Similarly, the TC analyses (N = 71) demonstrated a large effect size for verum pre- to post- (Δ = −13.6; *t* = 8.07; *p* =< 0.0001; *d* = 1.33), while sham had a smaller effect size (Δ = −11.9.1; *t* = 5.81; *p* =< 0.0001; *d* = 1.08).

- Intergroup Analyses

The ITT (N = 93) intergroup analyses comparing sham vs. verum showed a small effect size and was not statistically significant at mid-treatment (Δ = −1.1; *t* = 0.42; *p* = 0.67; *d* = 0.10). The intergroup analyses comparing sham vs. verum also showed a small effect size and was not statistically significant at the end of treatment (Δ = −2.1; *t* = 0.66; *p* = 0.51; *d* = 0.16). The intergroup TC analyses (N = 71) also showed a small effect size and were not statistically significant at both mid-treatment (Δ = −0.99; *t* = 0.36; *p* = 0.72; *d* = 0.09) and the end of treatment (Δ = −2.4; *t* = 0.76; *p* = 0.45; *d* = 0.19)

Pittsburgh Sleep Quality Index (PSQI) Severity (Table 4).

- Intragroup Analyses

The ITT analyses (N = 93) showed a moderate effect size of verum pre- to post- (Δ = −2.7; *t* = 4.47; *p* =< 0.0001; *d* = 0.73). The sham pre- to post- had a similar, moderate effect size (Δ = −2.6; *t* = 2.15; *p* = 0.04; *d* = 0.39). Similarly, the TC analyses (N = 71) also showed a moderate effect size of verum pre- to post- (Δ = −2.6; *t* = 4.51; *p* =< 0.0001; *d* = 0.72). The sham pre- to post- had a smaller effect size (Δ = −1.2.1; *t* = 2.30; *p* = 0.03; *d* = 0.40).

- Intergroup Analyses

In contrast to intragroup analyses, the ITT (N = 93) intergroup analyses comparing sham vs. verum showed a small effect size and were not statistically significant at mid-treatment (Δ = −0.63; *t* = 0.70; *p* = 0.48; *d* = 0.16). The intergroup analyses comparing sham vs. verum also showed a small effect size and were not statistically significant at the end of treatment (Δ = −1.8; *t* = 1.88; *p* = 0.06; *d* = 0.44). The intergroup TC analyses (N = 71) also showed a small effect size and were not statistically significant at both mid-treatment (Δ = −0.53; *t* = 0.57; *p* = 0.58; *d* = 0.14) and the end of treatment (Δ = −1.6; *t* = 1.53; *p* = 0.13; *d* = 0.37).

Safety: There were 64 AEs and no SAEs. In total, 7 AEs were related, 3 were possibly related, and 54 were unrelated to the study. Of those related, three had anxiety during physiology testing, one had nocturnal panic attacks, one had increased suicidal ideation that was mild and resolved, one withdrew pre-randomization due to increased symptoms, and one withdrew for intervention-related symptoms. No participants were withdrawn due to suicidal/homicidal ideation.

## 4. Discussion

### 4.1. Principal Findings

In this study designed to assess the impact of verum vs. sham acupuncture for veterans with combat-PTSD, post hoc analyses were conducted to assess the impact of these interventions on anxiety, depression, and insomnia. Analyses showed that both verum and sham acupuncture had moderate to large effect sizes on improving anxiety, depression, and insomnia symptoms, supporting the primary first part of this study’s hypothesis. However, intergroup differences were not statistically significant, and effect sizes were mild to moderate between verum and sham groups, refuting the second part of this study’s hypothesis. Of note, in ITT analyses for insomnia, verum acupuncture had a moderate effect size (*d* = 0.44) compared to sham acupuncture at the end of treatment, which was trending toward statistical significance (*p* = 0.06). The safety of acupuncture when delivered by competent practitioners has been established and is corroborated here with no serious adverse events or known physical injury from needling. Fidelity to protocol was also established.

### 4.2. Results in the Context of Other Research

Sham acupuncture was also associated with a significant, moderate to large reduction in anxiety, depression, and insomnia symptoms, which were not statistically different from verum acupuncture. However, in all three clinical conditions, verum trended toward larger effects. These data support previous work and reaffirm the notion that sham acupuncture can be a very powerful placebo. For clinicians, it is comforting and helpful to know that all types of acupuncture (i.e., both verum and sham) may be used as an adjunctive therapy with conventional medicine for anxiety, depression, and insomnia.

In contrast, sham and placebo procedures continue to be among the greatest challenges for acupuncture research since they are not fully inactive [27]. There are currently three types of sham approaches: insertion with manipulation at non-acupoints, superficial insertion absent further manipulation at verum acupoints, and non-insertive Streitberger and Park devices that adhere to the skin and utilize a blunt tip telescoping mechanism to retract without puncture [27,28]. Both insertive and non-insertive procedures may influence expectation, sensation, and contextualization, and both have shown effects in brain areas controlling sensation, cognition, and affect [29]. Research, however, has shown clinical differences between verum ACU and minimal needling techniques [30,31]. Other potential contributing factors accounting for the effects of sham conditions include clinician–patient interactions and the use of an optimal healing environment for participants.

Another potential explanation may lie within the theory of the specificity of acupoints. In accordance with traditional Chinese medicine pattern diagnosis, patients with anxiety, depression, and insomnia may have different pattern diagnoses than those with PTSD, which may result in very different sets of acupoints [32]. Since the current verum acupoints protocols for this study were developed specifically for PTSD, it is possible that the acupoints in this study were less effective in treating patients with anxiety, depression, and anxiety, thereby lessening the true effect sizes of verum acupuncture.

This study demonstrated that both verum and sham acupuncture had higher retention and satisfaction rates compared to approximately 50% treatment retention in a recent psychotherapy clinical trial for PTSD [3]. In terms of healthcare policy for the treatment of anxiety, depression, and insomnia, acupuncture may represent a safe, effective, and durable treatment in addition to conventional psychotherapies and medications.

### 4.3. Strengths and Limitations

The primary study strength was using a randomized, controlled, two-arm, single-blind trial to assess the potential nonspecific effects of acupuncturists’ attentiveness and of minimal—and supposed “placebo”—needling. Although the acupuncturists were not blind to intervention, fidelity video assessment suggested that the participants were. This design enhanced the internal validity and better assessed the specific verum acupuncture effect compared with minimal needling. Another strength is having standardized acupoints with a set of pre-selected elective acupoints, which is in agreement with the practices of consultant acupuncturists, supporting community standards and the reproducibility of this protocol by others [30]. This standard approach was developed in earlier work using multiple methods [32] and might be considered the gold standard for acupuncture research.

The primary study weakness was the lack of adequate power to detect statistical differences between verum and sham acupuncture groups for anxiety, depression, and insomnia. Since this study was powered for the effect on PTSD using previous acupuncture PTSD data, we could not perform adequate power analyses for other outcomes and justify a larger sample size than the total budget and scope that were funded. However, future studies evaluating the impact of acupuncture on anxiety, depression, and insomnia may be able to overcome this limitation by focusing the sample size calculations for only one specific condition.

Other potential limitations include the inability to conduct a double-blind intervention, the lack of an inert placebo, the variability of treatment approaches, the rare assessment of protocol adherence, and the inability to assess nonspecific factors on the treatment outcome. The current study was designed to have at least two practitioners, control specific and nonspecific behaviors, and assess protocol adherence, which is similar to current psychotherapy research [3]. Nonetheless, the acupuncturists were not blinded, there was undoubtedly practitioner variability, and the question of “best placebo control” for acupuncture remains. Lastly, the sponsor did not allow for a longer follow-up period to assess effect durability. Previously published data from this study found durability at three-months post-treatment for PTSD [16]. To better assess the durability of acupuncture treatment, a 6-month or 12-month post treatment follow-up would yield a stronger study design.

## 5. Conclusions: Toward Implementation and Research

Both verum and sham acupuncture showed moderate to large effect sizes in improving symptoms of anxiety, depression, and insomnia. Although verum and sham acupuncture did not differ in their effectiveness, acupuncture represents an effective and safe patient-center treatment option for patients who want to add this treatment in addition to conventional psychotherapies and medications. These findings call for more research about the potential synergy of acupuncture as an adjunctive therapy for current best practices, the durability of treatment, and the generalizability to both veteran and civilian populations.

## Figures and Tables

**Table 1:** Bivariate and Univariate Associations of Characteristics for Randomized Study Participants by Group.

			Group			
	Population		Verum		Sham	
	N	Mean, SD	N	Mean, SD	N	Mean, SD
Clinical Characteristics						
Hamilton Anxiety Rating Scale (HAMA)	93	32.8, 9.5	47	33.6, 9.2	46	32.0, 9.7
Beck’s Depression Inventory (BDI)	92	31.4, 10.9	46	32.0, 11.0	46	30.7, 11.0
Pittsburgh Sleep Quality Index (PSQT)	93	14.2, 3.5	47	14.2, 3.7	46	14.2, 3.3
Clinician-Administered PTSD Scale-5 (CAPS-5)	92	36.9, 6.3	47	37.1, 6.7	45	36.6, 5.8
	N	(%)	N	(%)	N	(%)
Age Group						
24–29	11	(11.8)	3	(6.4)	8	(17.4)
30–34	22	(23.7)	10	(21.3)	12	(26.1)
35–39	22	(23.7)	13	(27.7)	9	(19.6)
40–44	9	(9.7)	6	(12.8)	3	(6.5)
45–49	16	(17.2)	8	(17.0)	8	(17.4)
50+	13	(14.0)	7	(14.9)	6	(13.0)
Sex						
Male	85	(91.4)	43	(91.5)	42	(91.3)
Female	8	(8.6)	4	(8.5)	4	(8.7)
Country of Birth						
USA	72	(77.4)	35	(74.5)	37	(80.4)
Other	21	(22.6)	12	(25.5)	9	(19.6)
Education Level						
HS/GED	19	(20.4)	12	(25.5)	7	(15.2)
Some College	39	(41.9)	18	(38.3)	21	(45.7)
College Degree	35	(37.6)	17	(36.2)	18	(39.1)
Marital Status						
Married	46	(49.5)	24	(51.1)	22	(47.8)
Single	20	(21.5)	13	(27.7)	7	(15.2)
Divorced	21	(22.6)	8	(17.0)	13	(28.3)
Other	6	(6.5)	2	(4.3)	4	(8.7)
Religion						
Buddhist	4	(4.3)	3	(6.4)	1	(2.2)
Christian	46	(49.5)	22	(46.8)	24	(52.2)
Muslim	2	(2.2)	0	0	2	(4.3)
Other	21	(22.6)	10	(21.3)	11	(23.9)
None	20	(21.5)	12	(25.5)	8	(17.4)
Live With Self						
No	64	(68.8)	33	(70.2)	31	(67.4)
Yes	29	(31.2)	14	(29.8)	15	(32.6)
Live With Spouse						
No	49	(52.7)	24	(51.1)	25	(54.3)
Yes	44	(47.3)	23	(48.9)	21	(45.7)
Live With Children						
No	62	(66.7)	31	(66.0)	31	(67.4)
Yes	31	(33.3)	16	(34.0)	15	(32.6)
Live With Parents						
No	78	(83.9)	37	(78.7)	41	(89.1)
Yes	15	(16.1)	10	(21.3)	5	(10.9)
Live With Relatives						
No	86	(92.5)	44	(93.6)	42	(91.3)
Yes	7	(7.5)	3	(6.4)	4	(8.7)
Live With Others						
No	79	(84.9)	41	(87.2)	38	(82.6)
Yes	14	(15.1)	6	(12.8)	8	(17.4)
Annual Income						
Missing	1	(1.1)	1	(2.1)	0	0
USD 0–19,999	13	(14.0)	3	(6.4)	10	(21.7)
USD 20,000–34,999	14	(15.1)	6	(12.8)	8	(17.4)
USD 35,000–49,999	22	(23.7)	13	(27.7)	9	(19.6)
>USD 50,000	43	(46.2)	24	(51.1)	19	(41.3)
Employed						
No	51	(54.8)	30	(63.8)	21	(45.7)
Yes	42	(45.2)	17	(36.2)	25	(54.3)
Work Hours						
<40	16	(17.2)	6	(12.8)	10	(21.7)
40	22	(23.7)	12	(25.5)	10	(21.7)
>40	7	(7.5)	1	(2.1)	6	(13.0)
Missing	48	(51.6)	28	(59.6)	20	(43.5)
Race						
American Indian/Alaskan Native	2	(2.2)	2	(4.3)	0	0
White	44	(47.3)	23	(48.9)	21	(45.7)
Asian	17	(18.3)	8	(17.0)	9	(19.6)
Black/African American	12	(12.9)	5	(10.6)	7	(15.2)
More than one	15	(16.1)	6	(12.8)	9	(19.6)
Unknown	3	(3.2)	3	(6.4)	0	0
Ethnicity						
Hispanic	43	(46.2)	23	(48.9)	20	(43.5)
Non-Hispanic	50	(53.8)	24	(51.1)	26	(56.5)
Combat Exposure						
Light	3	(3.2)	1	(2.1)	2	(4.3)
Moderate Light	16	(17.2)	8	(17.0)	8	(17.4)
Moderate	31	(33.3)	16	(34.0)	15	(32.6)
Moderate Heavy	28	(30.1)	14	(29.8)	14	(30.4)
Heavy	15	(16.1)	8	(17.0)	7	(15.2)
Deployment Preparedness						
Low Preparedness	7	(7.5)	4	(8.5)	3	(6.5)
Moderate Preparedness	46	(49.5)	23	(48.9)	23	(50.0)
High Preparedness	40	(43.0)	20	(42.6)	20	(43.5)

There were no meaningful group differences on any variable in Table 1. Completer analysis (*n* = 71) also found no meaningful group differences on any variable in Table 1.

**Table 2:** Hamilton Anxiety Scores, Grouped by Time, Intention-to-Treat, and Treatment Completers.

		Pre-Treatment	Mid-Treatment	Post-Treatment
	N	Mean (SD)	Mean (SD)	Mean (SD)
Intention to Treat ***				
Total Randomized	93	32.8 (9.5)	N = 74, 23.1 (10.6)	N = 72, 21.1 (11.2)
Verum *	47	33.6 (9.2)	N = 38, 23.3 (10.8)	N = 39, 19.4 (10.3)
Sham *	46	32.0 (9.7)	N = 36, 22.9 (10.6)	N = 33, 23.2 (12.1)
Treatment Completers ****				
Total Sample	71	33.1 (9.6)	N = 69, 23.0 (10.7)	N = 68, 20.6 (11.1)
Verum **	39	33.4 (9.7)	N = 37, 22.9 (10.8)	N = 38, 18.8 (9.7)
Sham **	32	32.8 (9.5)	N = 32, 23.1 (10.7)	N = 30, 22.8 (12.5)

* ITT (N = 93); **intra-group**: Verum Pre to Post Δ = 14.1 (10.6); *d* = 1.3; *t* = 8.32; *p* < 0.0001. Sham Pre to Post Δ = 8.9 (9.6); *d* = 0.93; *t* = 5.33; *p* < 0.0001. ** Treatment completers (N = 71); **intra-group**: Verum Pre to Post Δ = 14.6 (10.2); *d* = 1.44; *t* = 8.85; *p* < 0.0001. Sham Pre to Post Δ = 9.9 (8.6); *d* = 1.15; *t* = 6.30; *p* < 0.0001. *** ITT (N = 93); inter-group: mid-treatment Δ = 0.35 (10.7); *d* = 0.03; *t* = 0.14; *p* = 0.89; end-treatment Δ = 3.8 (11.2); *d* = 0.34; *t* = 1.43; *p* = 0.16. **** Treatment completers (N = 71); inter-group: mid-treatment Δ = 0.21 (10.7); *d* = 0.02; *t* = 0.08; *p* = 0.94; end-treatment Δ = 4.0 (11.0); *d* = 0.36; *t* = 1.47; *p* = 0.15. “*d*” = Cohen *d*. “Tx” = Treatment. “Verum” = experimental group. “Sham” = placebo control group. Non-statistically significant time by treatment interaction based on repeated measures ANOVA; *p* = 0.37.

**Table 3:** Beck’s Depression Inventory Scores, Grouped by Time, Intention-to-Treat, and Treatment Completers.

		Pre-Treatment	Mid-Treatment	Post-Treatment
	N	Mean (SD)	Mean (SD)	Mean (SD)
Intention to Treat ***				
Total Sample	92	31.4 (10.9)	N = 74, 21.1 (11.3)	N = 71, 18.5 (13.0)
Verum *	46	32.0 (11.0)	N = 38, 20.5 (10.7)	N = 39, 17.5 (11.9)
Sham *	46	30.7 (11.0)	N = 36, 21.6 (12.0)	N = 32, 19.6 (14.4)
Treatment Completers ****				
Total Sample	70	31.0 (11.3)	N = 69, 20.6 (11.3)	N = 67, 18.1 (13.0)
Verum **	38	30.6 (11.2)	N = 37, 20.2 (10.6)	N = 38, 17.0 (11.5)
Sham **	32	31.6 (11.7)	N = 32, 21.2 (12.3)	N = 29, 19.4 (14.9)

* ITT (N = 93); **intra-group**: Verum Pre to Post Δ = 13.2 (10.4); *d* = 1.26; *t* = 7.80; *p* < 0.0001. Sham Pre to Post Δ = 11.5 (10.8); *d* = 0.93; *t* = 6.01; *p* < 0.0001. ** Treatment completers (N = 71); **intra-group**: Verum Pre to Post Δ = 13.6 (10.2); *d* = 1.33; *t* = 8.07; *p* < 0.0001. Sham Pre to Post Δ = 11.9 (11.1); *d* = 1.08; *t* = 5.81; *p* < 0.0001. *** ITT (N = 93); inter-group: mid-treatment Δ = 1.1 (11.3); *d* = 0.10; *t* = 0.42; *p* = 0.67; end-treatment Δ = 2.1 (13.1); *d* = 0.16; *t* = 0.66; *p* = 0.51. **** Treatment completers (N = 71); inter-group: mid-treatment Δ = 0.99 (11.4); *d* = 0.09; *t* = 0.36; *p* = 0.72; end-treatment Δ = 2.4 (13.1); *d* = 0.19; *t* = 0.76; *p* = 0.45. “*d*” = Cohen *d*. “Tx” = Treatment. “Verum” = experimental group. “Sham” = placebo control group. Non-statistically significant time by treatment interaction based on repeated measures ANOVA; *p* = 0.61.

**Table 4:** Pittsburgh Sleep Quality Index Scores, Grouped by Time, Intention-to-Treat, and Treatment Completers.

		Pre-Treatment	Mid-Treatment	Post-Treatment
	N	Mean (SD)	Mean (SD)	Mean (SD)
Intention to Treat ***				
Total Sample	93	14.2 (3.5)	N = 74, 13.2 (3.8)	N = 72, 12.0 (4.2)
Verum *	47	14.2 (3.7)	N = 38, 12.9 (4.1)	N = 39, 11.2 (4.4)
Sham *	46	14.2 (3.3)	N = 36, 13.5 (3.6)	N = 33, 13.0 (3.8)
Treatment Completers ****				
Total Sample	71	14.0 (3.5)	N = 69, 13.0 (3.8)	N = 68, 11.8 (4.2)
Verum **	39	13.9 (3.8)	N = 37, 12.8 (4.1)	N = 38, 11.1 (4.5)
Sham **	32	14.1 (3.2)	N = 32, 13.3 (3.4)	N = 30, 12.7 (3.8)

* ITT (N = 93); **intra-group**: Verum Pre to Post Δ = 2.7 (3.7); *d* = 0.73; *t* = 4.47; *p* < 0.0001. Sham Pre to Post Δ = 1.2 (3.1); *d* = 0.39; *t* = 2.15; *p* = 0.04. ** ITT (N = 93); **intra-group**: Verum Pre to Post Δ = 2.6 (3.7); *d* = 0.72; *t* = 4.51; *p* < 0.0001. Sham Pre to Post Δ = 1.2 (3.0); *d* = 0.40; *t* = 2.30; *p* = 0.03. *** ITT (N = 93); inter-group: mid-treatment Δ = 0.63 (3.9); *d* = 0.16; *t* = 0.70; *p* = 0.48; end-treatment Δ = 1.8 (4.1); *d* = 0.44; *t* = 1.88; *p* = 0.06. **** ITT (N = 93); inter-group: mid-treatment Δ = 0.53 (3.8); *d* = 0.14; *t* = 0.57; *p* = 0.58; end-treatment Δ = 1.6 (4.2); *d* = 0.37; *t* = 1.53; *p* = 0.13. “*d*” = Cohen *d*. “Tx” = Treatment. “Verum” = experimental group. “Sham” = placebo control group. Non-Statistically significant time by treatment interaction based on repeated measures ANOVA. *p* = 0.08.

## Data Availability

Data sharing will be considered under negotiation between interested partners and authors with VA IRB participation. Requests should be made to the corresponding author.
